# Three‐dimensional, PEG‐based hydrogels induce spheroid formation and enhance viability of A2058 melanoma cells

**DOI:** 10.1002/2211-5463.13719

**Published:** 2023-10-30

**Authors:** Kata Nóra Enyedi, Gábor Enyedi, Eszter Lajkó

**Affiliations:** ^1^ Faculty of Science, Institute of Chemistry Eötvös Loránd University Budapest Hungary; ^2^ Department of Organic Chemistry, ELKH‐ELTE Research Group of the Peptide Chemistry Institute Eötvös Loránd University Budapest Hungary; ^3^ Department of Research and Development En‐Co Software Zrt. Budapest Hungary; ^4^ Department of Genetics, Cell and Immunobiology Semmelweis University Budapest Hungary

**Keywords:** artificial intelligence, cancer, hydrogel, melanoma, polyethylene glycol, spheroids

## Abstract

Traditional drug screening methods use monolayer (2D) tumor cell cultures, which lack basic features of tumor complexity. As an alternative, 3D hydrogels have begun to emerge as simple, time‐, and cost‐saving systems. One of the most promising candidates, synthetic alkoxysilane‐PEG (polyethylene glycol)‐based hydrogels, are formed by “sol–gel” polymerization in an aqueous medium, which allows control over the incorporated elements. Our aims were to optimize siloxane‐PEG hydrogels for three different cell lines of skin origin and utilize these 3D hydrogels as a feasible drug (e.g., daunorubicin) screening assay. A drastic increase in survival and the formation of cellular aggregates (spheroids) could be observed in A2058 melanoma cells, but not in keratinocyte and endothelial cell lines. A deep‐learning neural network was trained to recognize and distinguish between the cellular formations and allowed the fast processing of hundreds of microscopic images. We developed an artificial intelligence (AI)‐assisted application (https://github.com/enyecz/CancerDetector2), which indicated that, in terms of average area of the spheroids treated with daunorubicin, A2058 melanoma cell 3D aggregates have better survival in a hydrogel containing 15% bis‐mono‐ethoxysilane‐PEG.

## Graphical abstract

Our aim was to utilize siloxane‐polyethylene glycol (PEG) hydrogels as a feasible viability assay against daunorubicin. The formation of cellular aggregates (spheroids) could be observed in melanoma cells but not in keratinocyte and endothelial cell lines. An artificial intelligence (AI)‐assisted application that we developed indicated that melanoma cell spheroids treated with daunorubicin have better survival in a hydrogel containing 15% bis‐mono‐ethoxysilane‐PEG.

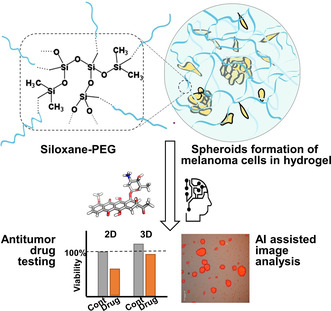

**Abbreviations**
2D: two‐dimensional
3D: three‐dimensional
AI: artificial intelligence
Dau: daunorubicin
NaF: sodium fluoride
PEG: polyethylene glycol
SD: standard deviation
v/v%: volume/volume %

As the numbers of cancer patients increase worldwide, both pharmaceutical companies and the scientific community try to invest more into the development of novel chemotherapeutic drugs [1]. During the early stages, monolayer (two‐dimensional, 2D) cell cultures are used for rapid biological screening assays. However, this configuration limits the complex cellular interactions that are present in the multilayer structure of a tumor and cannot represent the *in vivo* milieu. Often, due to these misleading preclinical *in vitro* models, many anticancer candidates fail long before clinical trials [2].

Over the last decade, several sophisticated culture platforms were developed to mimic the tumor niche (e.g., microbioreactors) in an *in vitro* setting [3, 4]. The major issue with these methods is the highly complicated experimental setup, which requires expertise and expensive maintenance. As an alternative, three‐dimensional (3D) hydrogels have begun to emerge as simpler, time‐, and cost‐saving systems.

Commercially available hydrogels are usually biomaterial‐based (e.g., Matrigel, collagen). Although they are theoretically ideal for 3D cell cultures, the tricky handling and varying lot‐to‐lot qualities do not make them suitable for high‐throughput screening of drugs. The question of reproducibility is a general problem with hydrogels of biological origin [5]. However, in this respect, synthetic 3D systems may provide alternative models for *in vitro* assays, since the qualitative and quantitative composition of the synthetic materials can be controlled.

Polyethylene glycol (PEG) is one of the most common synthetic building blocks for hydrogels due to their high hydrophilicity and biological inertness [6]. PEG‐based hydrogels, therefore, enable controlled and defined investigation of cellular behavior (e.g., proliferation, viability, and migration). There are several types of PEG polymerization, but they typically require complex functionalization of the building blocks and elaborate reactions for the network formation [7, 8, 9]. However, a more straightforward method was developed earlier by Echalier *et al*. [10]. The hydrogel is based on the “sol–gel” polymerization of silylated‐PEG in cell‐culture medium and showed promising results in culturing chondrocyte stem cells. It has the advantage of a simple monomer synthesis and a biocompatible and reproducible gel formation, which is crucial in 3D cellular assays.

Despite the favorable properties of the aforementioned hydrogel, it was questionable whether this gel would be suitable for culturing melanoma cells, one of the most studied tumor types, and other skin related, but normal cells such as keratinocytes or microvascular endothelial cells, which may contribute to melanoma microenvironment. The 3D spatial arrangement of cells is crucial in the pathobiology of the melanoma as well as in the response to antitumor agent [11]. It is also an important aspect that the PEG‐based hydrogels could be utilized as an efficient 3D model to test different kind of drugs (e.g., chemotherapeutics). Therefore, we aimed to investigate: (a) the cellular behavior of cancer cells in this silylated‐PEG hydrogel, (b) if loosening the polymer network (slightly increasing its average mesh size) would result in a better matrix for cancer cells, and (c) the applicability of this hydrogel as a feasible viability assay against a chemotherapeutic drug to test and compare the cellular responses.

In order to answer these questions, three cell lines of skin origin were chosen (A2058 human melanoma, HaCaT human keratinocyte, and HMEC‐1 human microvascular endothelial cell lines). Several PEG‐based hydrogels with increasing mesh size (“loosened network”) were synthetized and used as 3D model matrices, against a clinically used chemotherapeutic agent, daunorubicin (Dau). We chose Dau, an anthracycline‐type chemotherapeutic agent, since its efficacy is affected by dose‐limiting side‐effects (e.g., cardiomyopathy) and drug‐resistance that can emerge easily [12]. Our present results demonstrated that in some of the PEG‐based hydrogels, A2058 melanoma cells—but not “normal” cells—showed increased resistance to Dau and also preferred to form cellular aggregates.

Since different cell aggregates and other cellular structures, which are more likely to form in hydrogels, can contribute to drug resistance, we also aimed to simplify the analysis of cellular morphology and aggregate formation in such gels. Z‐stacking is a commonly used method in microscopic analysis for 3D systems. However, it can be time‐consuming and requires instruments capable of Z‐stacking; therefore, it is not compatible with examining a wider sample size. Artificial intelligence (AI) supported recognition and processing can be an option in such cases. Therefore, in this project, we also aimed to design a neural network‐based application (https://github.com/enyecz/CancerDetector2), which uses only simple microscopic images for analysis.

## Materials and methods

Unless otherwise noted, all supplies were purchased from Thermo Fisher Scientific (Waltham, MA, USA). Daunorubicin was a kind gift from Dr. Gábor Mező (ELKH‐ELTE Research Group of Peptide Chemistry, Budapest, Hungary).

### Synthesis of alkoxy‐silylated PEG monomer

Bis‐triethoxy‐sylilated‐PEG and bis‐dimethyl‐monoethoxy‐sililated PEG monomers were prepared as earlier reported [13, 14]. Full description of the monomer synthesis can be found in Appendix S1.

### Cell lines and culture conditions

Human melanoma cell line A2058, obtained from the European Collection of Authenticated Cell Cultures (ECACC, Salisbury, UK), was cultured in RPMI 1640 medium supplemented with 10% (v/v %) fetal bovine serum (FBS, Biosera Europe, Nuaillé, France), 1% l‐glutamine, and 1% penicillin/streptomycin (Gibco, Invitrogen Corporation, New York, USA).

HaCaT (American Type Culture Collection, ATCC, Manassas, VA, USA) cells were cultured in DMEM medium, supplemented with 10% FBS (Biosera Europe, Nuaillé, France), 1% l‐glutamine, 1% pyruvate, 1% penicillin/streptomycin (Gibco, Invitrogen Corporation, New York, USA), and 1% nonessential amino acid and 4500 mg·L^−1^ glucose.

Human microvascular endothelial cell line, HMEC‐1 (Invitrogen Life Technologies, Carlsbad, CA, USA), was cultured in MCDB‐131 medium, supplemented with 10% FBS (Biosera Europe, Nuaillé, France), 2% l‐glutamine, 1% penicillin/streptomycin (Gibco, Invitrogen Corporation, New York, USA), 1 μg·mL^−1^ hydrocortisone, and 10 ng·mL^−1^ EGF (epidermal growth factor). The T25 culture flask (Sarstedt AG & Co. KG, Nümbrecht, Germany) was coated previously with 0.02% gelatin solved in PBS (phosphate buffer saline, pH 7.2).

All cells were propagated in standard cell culture conditions (37 °C, 5% CO_2_).

### Hydrogel formation for *in vitro* measurements

Hydrogels were prepared 24 h prior to cell seeding. A 25 w/w % solution of the monomers in incomplete, serum‐free RPMI 1640 medium with 1% l‐glutamine, 1% penicillin/streptomycin was prepared and mixed in the appropriate molar ratio. Sodium fluoride (NaF) was added to the monomer mixture with a final concentration of 3 mg·mL^−1^ and the hydrogel‐solution was diluted to 20 w/w % final concentration. This was directly followed by the distribution of 50–50 μL of monomer solution into a 96‐well optical bottom plate and was incubated for 24 h. Before cell seeding, the coated wells were washed two times with incomplete medium and three times with serum containing, complete cell culture medium.

### Viability assay and fluorescence microscopic detection

To define the IC50 values of Dau in 2D monolayer culture, CellTiterGlo (Promega, Madison, WI, USA) viability reagent was used. A2058 cells were seeded at a density of 10 000 cells/well; HaCaT cells were seeded at a density of 5000 cells/well; and HMEC‐1 cells were seeded at a density of 20 000 cells/well in 50–50 μL FBS containing medium on a 96‐well plate with optical bottom. Cells were then incubated for 48 h and then 50 μL/well of fresh, serum‐containing medium was added. At 96 h of culturing, cells were treated and incubated with Dau (concentration range: 0.1–50 μm). After 24 h of treatment, 100–100 μL CellTiterGlo reagent was added to each well and the luminescence signal was measured by Fluoroskan FL Microplate Fluorometer and Luminometer (Thermo Scientific, Waltham, MA, USA).

For testing cell viability in 3D, after the gel formation and washing, 10 000 cells/well for A2058; 20 000 cells/well for HMEC‐1 and 5000 cells/well for HaCaT were seeded on the hydrogels in 50–50 μL serum‐containing medium. Cells were then incubated for 48 h followed by the addition of 50–50 μL of fresh, FBS‐containing medium, and another 48‐h culturing step. The 96‐h cultivation time for our PEG‐based hydrogels was chosen because a longer period of time was needed for the cells to infiltrate into the hydrogels. Furthermore, in recent publications comparing 2D and 3D cell culture models, cells were cultured for 72 h or even longer (e.g., 7 days) [15, 16]. At 96 h of culturing, cells were treated and incubated with Dau at its IC50 concentrations. After 24 h of treatment, cell viability in the hydrogels was assessed by the addition of 100–100 μL CellTiterGlo 3D (Promega, Madison, WI, USA), developed for 3D cultures, to each well. The luminescence signal was measured by Fluoroskan FL Microplate Fluorometer and Luminometer (Thermo Scientific, Waltham, MA, USA).

In case of measuring the cell viability in hydrogels, the cell viability data of the untreated cells were normalized to that of the 2D system and expressed as a percentage (Normalized live cell number in 2D is the base, 100%). When cell viability was evaluated after Dau treatment, the viable cell number in the Dau‐treated group was normalized to that of the corresponding untreated cells in each hydrogel type and 2D condition.

Images were captured (5 × Plan‐Apochromat λ/0.35 NA objective with 2 × tube lens) after treatment with Dau with Celldiscoverer 7 system (Zeiss, Jena, Germany) using the zen blue 2.6 software (Carl Zeiss AG, Jena, Germany).

### Application development

For the evaluation of the cellular structures formed in the gels, we developed an AI‐assisted application by using a deep‐learning neural network. The application is based on semantic image segmentation. It is a process where a semantic label is assigned to each of the pixels of a given image. The goal is to identify semantically meaningful areas: in our case, to identify pixels belonging to cellular aggregations and “other pixels”, that is the segments of the background. There are many solutions for such a task. First, we made experiments with Google's Deeplab v3+ [17] and Segformer [18], but the results were not sufficiently precise for our purpose. Finally, we found that the recent versions of the relatively simple YOLO network, both YOLOv5 and YOLOv8 (Ultralytics YOLOv5, available online: https://github.com/ultralytics/yolov5, Ultralytics YOLOv8, available online: https://github.com/ultralytics/ultralytics) perform very well. Note that YOLO has been in active development since 2015 by many large research groups and although it is using techniques slightly inferior to, for example, vision transformers such as Segformer, it is still an extremely sophisticated solution. The final source code doing complete image processing and its usage guide can be found at https://github.com/enyecz/CancerDetector2.

### Statistical analysis

Triplicates were used for each assay and each measurement was repeated with different batches of the hydrogel. All quantitative data are presented as mean ± standard deviation (SD).

The results were evaluated by MS Excel and originpro 2020 software (OriginLab Corporation, Northampton, MA, USA). In case of the viability measurements, the statistical significance was determined by one‐way analysis of variance (ANOVA) followed by Tukey's *post hoc* test. When analyzing the number of the spheroids and average area of spheroids, the levels of significance were determined by paired, two‐tailed *t*‐test if the values of a given data series were compared to the 0‐h time point. Unpaired two‐tailed *t*‐test was used to determine significance if at given time points the values of the two hydrogels were compared. The changes were considered statistically significant if *P*‐value is < 0.05.

## Results and Discussion

**Fig. 1:**
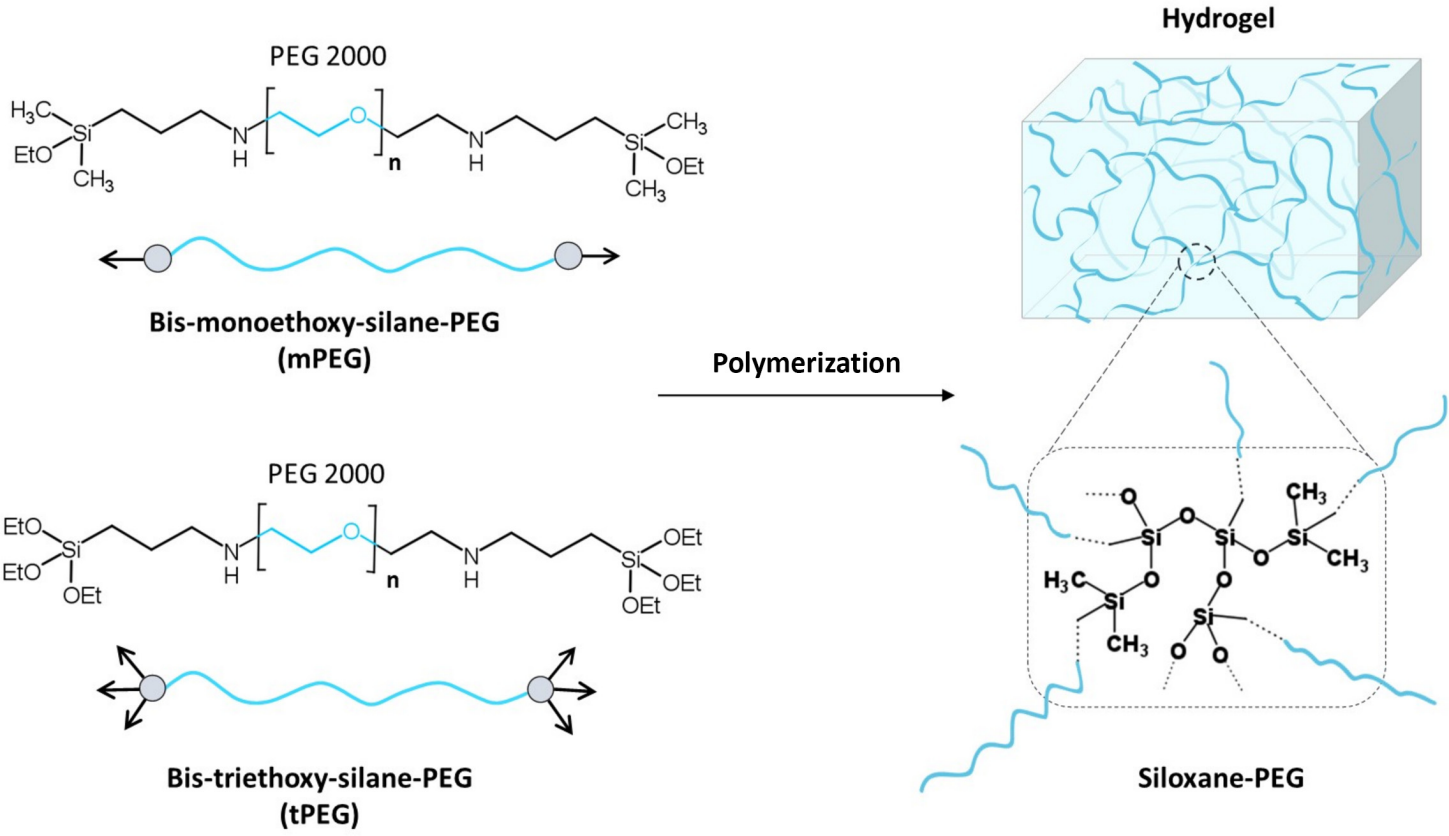
Hydrogels are formed using bis‐mono‐ethoxysilane‐ and bis‐triethoxysilane‐PEG (mPEG and tPEG).

The original hydrogel developed by Echalier *et al*. was intended for chondrocytes, but our goal was to optimize it for different cell lines representing tumor cells (A2058 cell line) or normal cells (HaCaT human keratinocytes [19] and HMEC‐1 human microvascular endothelial cells [20]) as well as apply it as a drug‐screening system [10]. It has become well‐known that different cell types prefer microenvironment with different mechanical properties (e.g., rigidity) [21] and their sensitivity to drugs (e.g., chemotherapeutics) depends on the surface characteristics [22]. Therefore, we expanded the hydrogel's average molecular weight by using bis‐mono and bis‐tri functionalized PEG derivatives (bis‐mono‐alkoxysilane‐mPEG; bis‐tri‐alkoxysilane derivatives‐tPEG) as building blocks (Fig. 1), hence increasing average mesh size in small steps.

This difference in the hydrogels also lead to changes in the hydrogel “scaffold” and consequently to different cellular behaviors. Different cell vitality and in case of A2058, morphological changes were also observed depending on the average molecular weight of the hydrogel network (mol% of mPEG).

### Influence of hydrogels on cell viability and survival

Cytotoxicity is the first‐line biological test for *in vitro* chemotherapeutic drug screening. Three cell lines of skin origin were chosen: A2058 (human melanoma), HaCaT (human keratinocytes), and HMEC‐1 (human dermal microvascular endothelial cells). First, the IC50 values of Dau—a clinically used chemotherapeutic drug—was determined for each cell line growing in monolayer (2D) (Figs S1–S3). In the next step, the cells were seeded on hydrogels containing different mol% of mPEG. Cells cultured on the plain surface of the optical‐bottom plate served as 2D condition, a basis for comparison. After reaching confluency (96 h) in monolayer culture, cells were treated with Dau at IC50 concentration, accordingly. It is known that the outer cells of a 3D culture reacts differently to a drug than the inner cells do [23]. This phenomenon raises the question how the amount of drug determined for cells cultured in a 2D substrate can be used for 3D cultures. Based on the literature [15, 16], treating the cells in PEG hydrogels with IC50 concentrations measured in 2D, is a well‐accepted experimental setup to demonstrate the difference between 2D and 3D cultures.

Overall, each cell line showed good cell viability in the matrices, which demonstrated that the hydrogels provided a suitable condition for cell cultures. However, prominent differences could be observed in terms of live/dead cell ratio after Dau treatment and in aggregate (spheroid) formation.

**Fig. 2:**
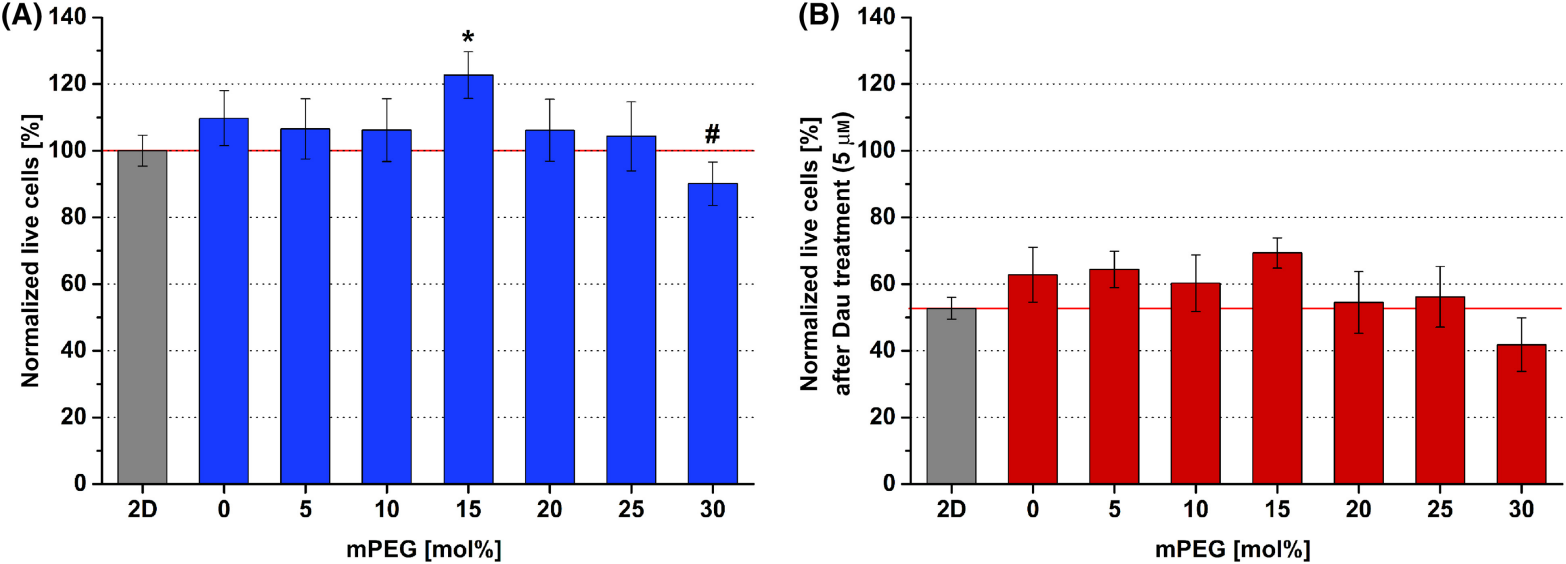
Viability of HaCaT cells in different hydrogels, cultured for 4 days, then treated with daunorubicin (Dau) for further 24 h. (A) Viability data of untreated cells in different hydrogel containing different mol% of mPEG (bis‐mono‐ethoxysilane‐PEG) were normalized to 2D growing cells (Normalized live cells = 100%). (B) Viability data of HaCaT cells treated with Dau (5 μm) were normalized to that of the corresponding non‐treated cells (Normalized live cells). Data are presented as mean values ± SD (*n* = 3). The levels of significance were determined by a one‐way ANOVA test with Tukey's *post hoc* test. Values of *P* < 0.05 were considered significant compared to 2D condition indicated by * asterisk and compared to 0% mPEG hydrogel indicated by ^#^.

In the case of HaCaT keratinocytes, neither 2D nor 3D hydrogels were preferred exclusively. The only significant difference was found in 15 mol% mPEG hydrogels. In case of the nontreated groups, ~ 20% more cells could be observed, compared with 2D control (Fig. 2A). However, this did not have any substantial effect on cell survival against Dau treatment (Fig. 2B).

**Fig. 3:**
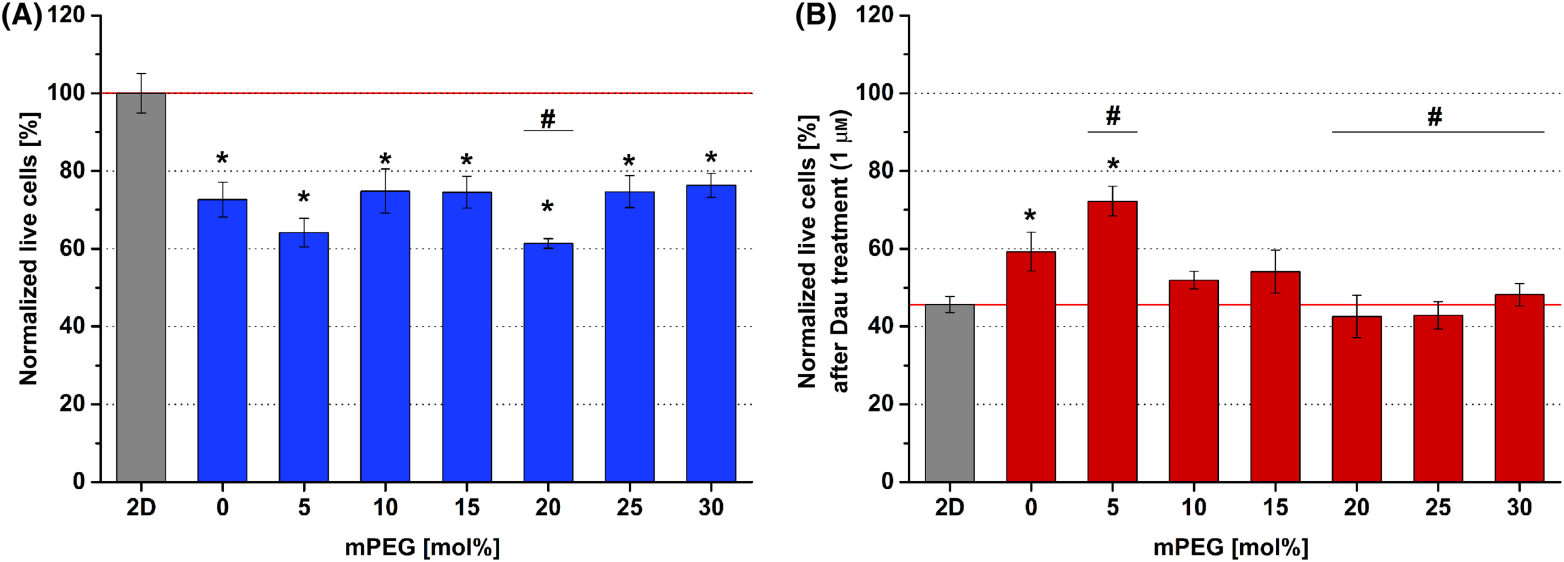
HMEC‐1 cell viability in different hydrogels, cultured for 4 days, treated with daunorubicin (Dau) for further 24 h. (A) Viability data of untreated cells in different hydrogel containing different mol% of mPEG (bis‐mono‐ethoxysilane‐PEG) were normalized to 2D growing cells (Normalized live cells = 100%). (B) Viability data of HMEC‐1 cells treated with Dau (1 μm) were normalized to that of the corresponding nontreated cells (Normalized live cells). Data are presented as mean values ± SD (*n* = 3). The levels of significance were determined by a one‐way ANOVA test with Tukey's *post hoc* test. Values of *P* < 0.05 were considered significant compared to 2D condition indicated by * asterisks and compared to 0% mPEG hydrogel indicated by ^#^.

HMEC‐1 endothelial cells showed less compatibility with the 3D system: Lower cell viability (~ 65–80%) was detected for the cells growing in the hydrogels than the cells cultured in 2D model (100%) (Fig. 3A). However, it is interesting to note, that in most of the hydrogels similar or even better (e.g., 5% mPEG), survival rate against Dau‐treatment could be observed, which would suggest that using an inert 3D system could be advantageous (Fig. 3B).

**Fig. 4:**
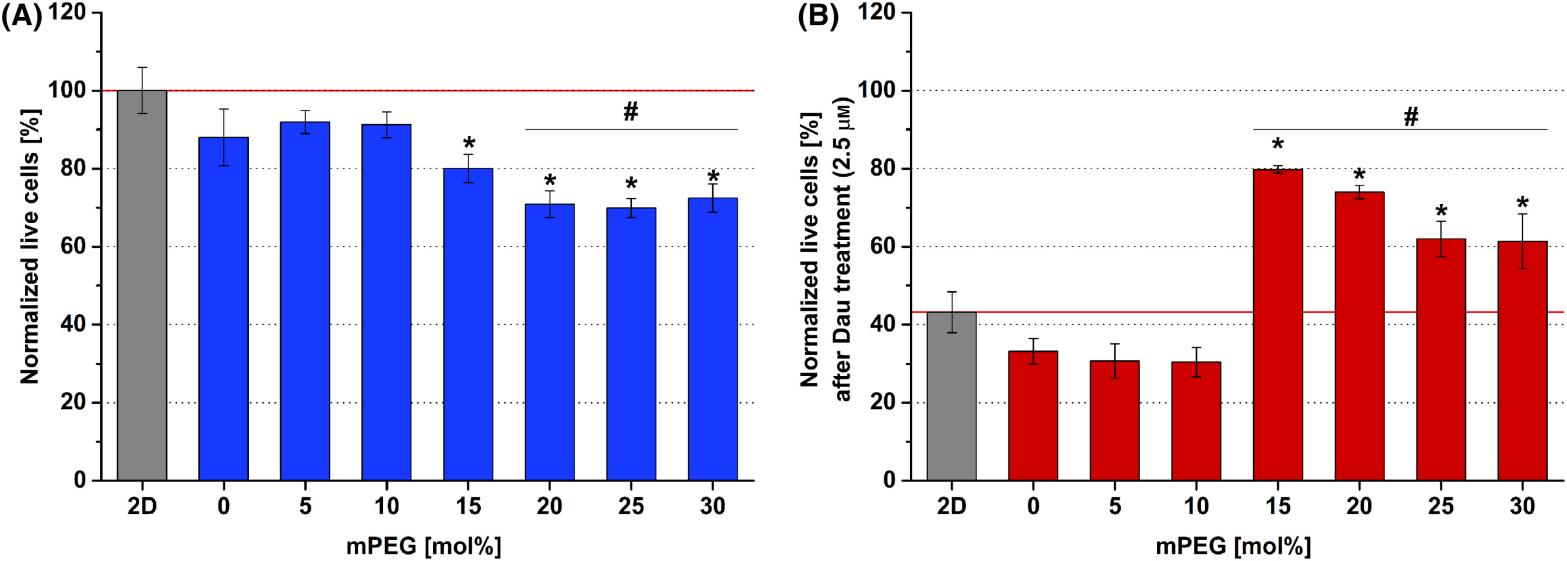
A2058 cell viability in different hydrogels, cultured for 4 days, treated with daunorubicin (Dau) for further 24 h. (A) Viability data of untreated cells in different hydrogel containing different mol% of mPEG (bis‐mono‐ethoxysilane‐PEG) were normalized to 2D growing cells (Normalized live cells = 100%). (B) Viability data of A2058 cells treated with Dau at IC50 (2.5 μm) were normalized to that of the corresponding nontreated cells (Normalized live cells). Data are presented as mean values ± SD (*n* = 3). The levels of significance was determined by a one‐way ANOVA test with Tukey's *post hoc* test. Values of *P* < 0.05 were considered significant compared to 2D condition indicated by * asterisks and compared to 0% mPEG indicated by ^#^.

A2058 human melanoma cell line showed very different behavior compared with the previously mentioned HaCaT and HMEC‐1 cell lines representing normal cells. In case of the untreated living cells, a slow decrease in the ratio of the living cells was detected comparing to the 2D control with the increasing concentrations of mPEG in hydrogels (Fig. 4A).

Like the other two cell lines, A2058 cells also tolerated the hydrogel matrices. A significant difference in the survival rate could be observed in case of the 15–20 mol% mPEG hydrogels, where the live/dead cell ratio was ~ 80–75% after Dau treatment, while in the 2D group, this was only ~ 42% (Fig. 4B). Unlike with the other tested cell lines, multilayered cell aggregates (ranging from 50 to 200 μm) could be detected in the hydrogels.

### Spheroid formations of A2058 cells

**Fig. 5:**
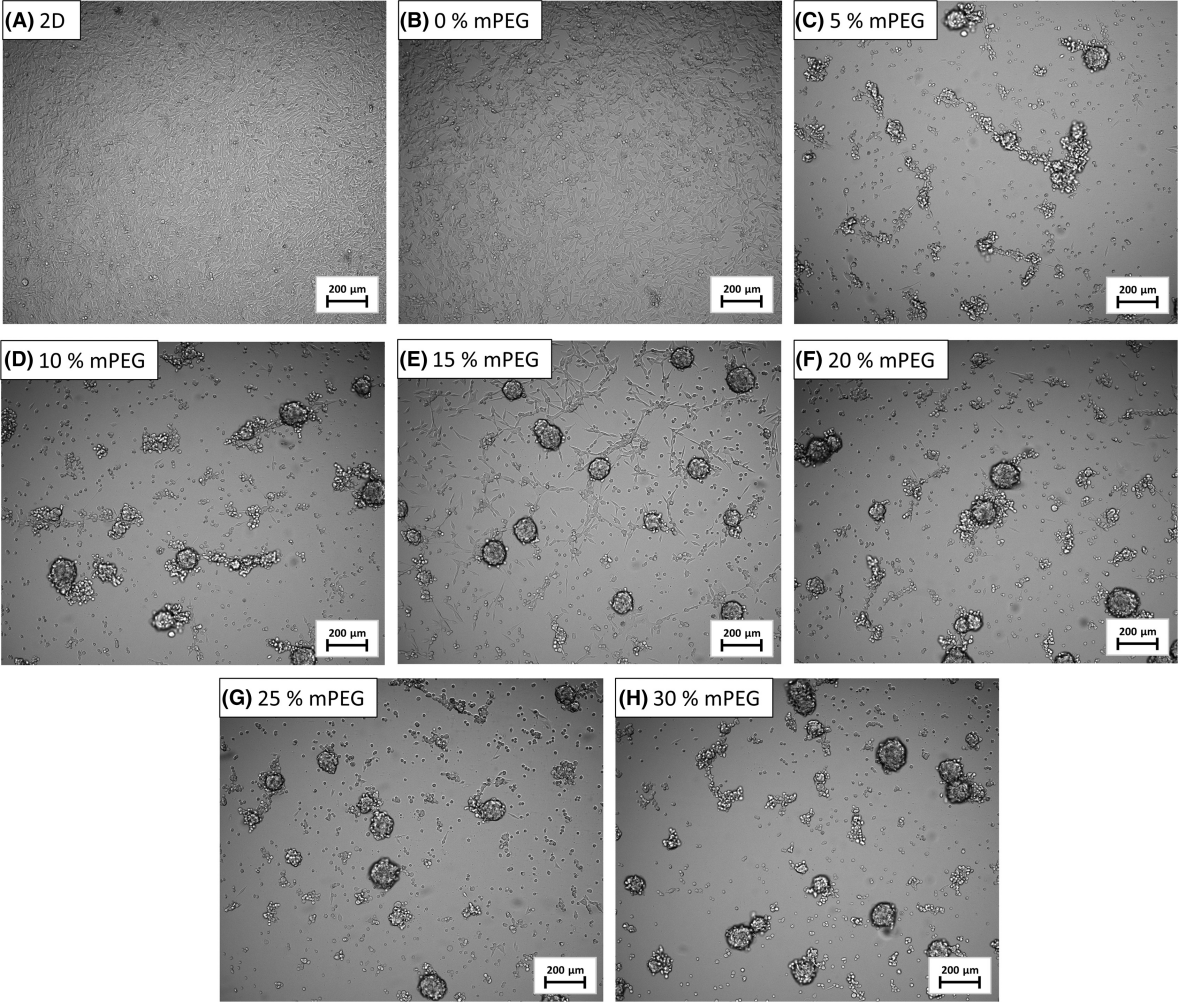
A2058 cells in (A) 2D and in hydrogels containing (B) 0, (C) 5, (D) 10, (E) 15, (F) 20; (G) 25 and (H) 30 mol% of mPEG (bis‐mono‐ethoxysilane‐PEG) 96 h after seeding. Images were captured by Celldiscoverer7 (5 × Plan‐Apochromat λ/0.35 NA objective with 2 × tube lens), the scalebar is 200 μm.

A2058 melanoma cell line showed the most prominent differences in hydrogel matrices, compared with 2D condition. In hydrogels containing 5–30 mol% mPEG, not only did the viability change, but elongated multicellular aggregates, called “spheroids” were also observed (Fig. 5). This phenomenon is not without precedent in the corresponding references: Spheroid formations of A2058 were reported earlier using different techniques, but not with PEG‐based hydrogels [24, 25]. Furthermore, these conventional spheroid formation techniques generally use a mechanical/magnetic/gravitational force none of which will provide a physiologically formed *in vitro* 3D model. Our system is unique as spheroid development is independent of these forces and both the individual cells and the spheroids coexist in the system.

### Effect of daunorubicin on A2058 aggregates

We hypothesized that the resulting spheroids are somehow related to the increased resistance of A2058 cells growing in hydrogels containing 15–30 mol% mPEG against Dau (Fig. 4B). Cellular toxicity was followed in more detail in the best‐ and the worst‐performing gels (hydrogels with 10% mPEG and 15% mPEG, respectively) to shed light on the essential difference between the two hydrogels. Therefore, images were taken in each hour after Dau treatment for both hydrogels (Videos S1 and S2).

**Fig. 6:**
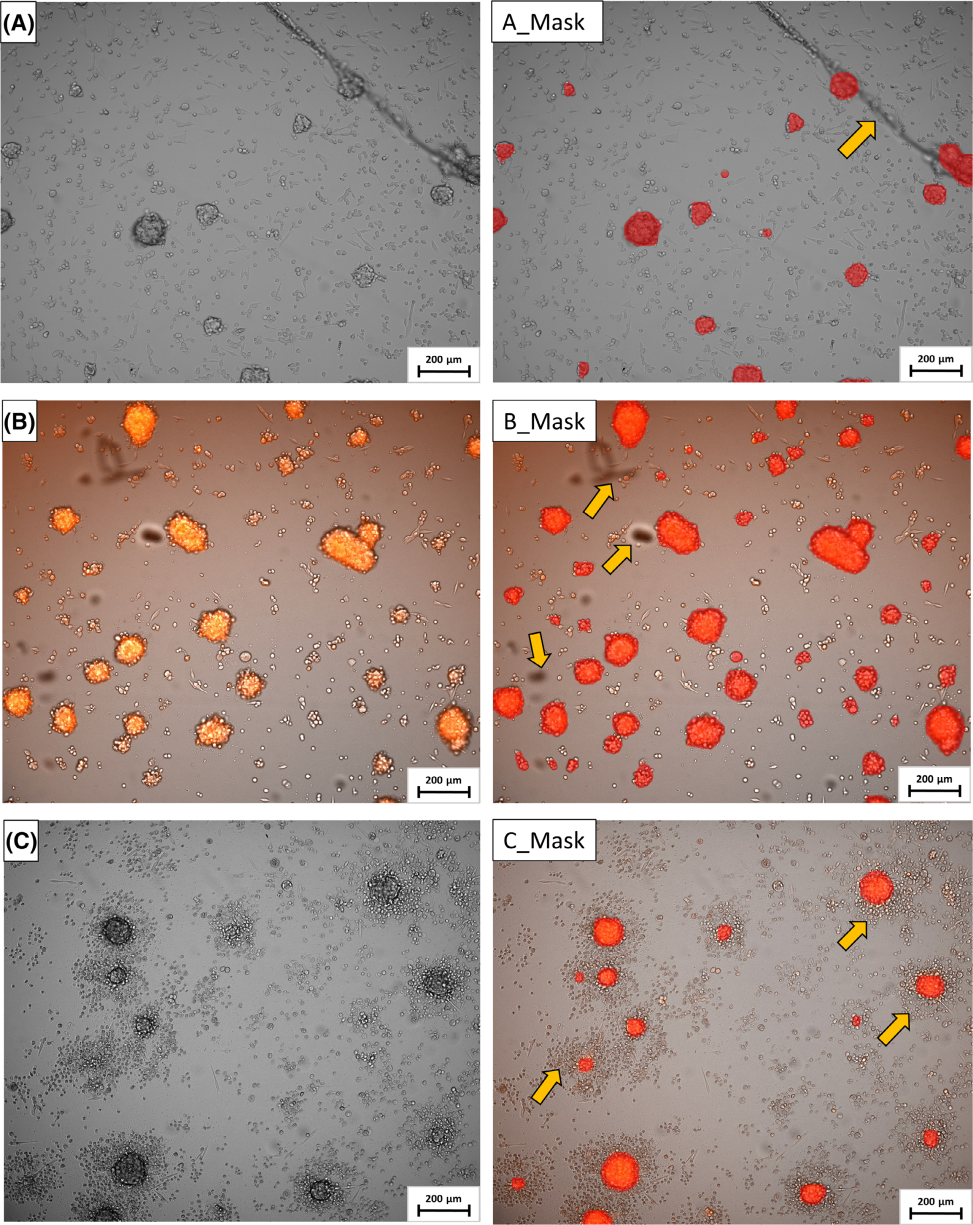
Example of spheroid recognition assisted by AI. Complicated images can be processed (e.g., spots and debris). Spheroids partly out of picture, small aggregates and cell debris (indicated by yellow arrows) can be excluded. (A–C) Original microscopic images (5 × Plan‐Apochromat λ/0.35 NA objective with 2 × tube lens) of control or daunorubicin‐treated A2058 cells; (A_Mask–C_Mask) AI processed pictures, accordingly. The scalebar is 200 μm.

Knowing the limitations (e.g., poor ability to discriminate small cell aggregates and spheroids among debris) of classical image analysis software (e.g., imagej), we developed a deep learning‐based AI‐based image analysis application to quantify the changes in the number and the dimensions/extent of spheroids after Dau treatment. The trained neural‐network can distinguish between debris/spots/uncommon shapes and small spheroids on microscopic images in our hydrogel system. The application even allows the exclusion of small aggregates and partly out‐of‐picture spheroids, this feature is adjustable (Fig. 6). One of the advantages of our application is that it enables the analysis of simple microscopic images; thus, the time‐consuming 3D (Z‐stacking) recordings could be replaced. This kind of image analysis gives albeit approximate, but a rapid image evaluation, which is especially advantageous in screening assays. The application is free to use and can be downloaded from https://github.com/enyecz/CancerDetector2.

**Fig. 7:**
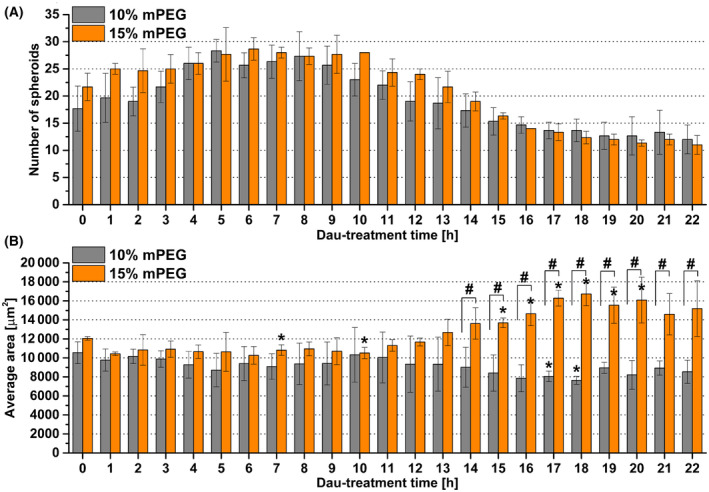
Image analysis of 10 and 15 mol% mPEG (bis‐mono‐ethoxysilane‐PEG) hydrogels after treatment with daunorubicin (Dau). Images were taken each hour and terminated when no significant changes can be registered further (22 h). (A) Number of spheroids and (B) average area of spheroids in 10% and 15% mPEG hydrogels after Dau treatment. Data are presented as mean values ± SD (*n* = 3). The levels of significance were determined by paired, two‐tailed *t*‐test if the values of a given data series were compared to the 0‐h time point. Unpaired two‐tailed *t*‐test was used to determine significance if at given time points the values of the two hydrogels were compared. Values of *P* < 0.05 were considered significant compared to 0 h indicated by * asterisks and between hydrogels indicated by ^#^.

Data acquired from the image analysis show that in the initial phase (0–5 h) of the Dau treatment, new small cellular clusters appeared in both hydrogels, presumably in response to treatment. As a result, the number of spheroids evened out in the two gels (5–9 h), but later (12–22 h) spheroids number declined due to the cytotoxicity of Dau (Fig. 7). These data should be considered along with the mean area of the spheroids. In the 4–10‐h time period after Dau treatment, there was an increase in the spheroid number in both of the gels, but the area did not change significantly in this time interval (Fig. 7A,B). Such an increase in the number of spheroids was not observed in the untreated groups in any of the gels (Figs S4A and S5A). However, the number of spheroids is almost the same in both gels through the whole time, but the average area highly increases later, especially after 12‐h treatment, in the optimal gel (15% mPEG). This would indicate that the remaining spheroids not only survived, but also increased their area probably due to cell proliferation or more remaining cells clustered into the spheroids (Fig. 7B; Fig. S5B). Comparing the untreated cells, there was no difference in the average area of spheroid between the two gels (Figs S4B and S5B). These results suggest that spheroids in 15% mPEG gel have an advantage in survival. This finding also confirms the general experience that spheroids can be more resistant to drugs than monolayer cell cultures [26, 27].

Nevertheless, it is important to note, that this analysis focused only on the changes in the multicellular aggregates and did not take into consideration the full number of individual cells.

## Conclusions

In the present study, we aimed to utilize a synthetic, PEG‐based 3D hydrogel that is easy to handle, compatible with wide variety of cells and can be used as a feasible and rapid viability assay against chemotherapeutic drug to test and compare the cellular responses. To this end, the hydrogels were optimized for three, different cell lines of skin origin representing tumor (A2058 melanoma cells) and normal cells (HaCaT and HMEC‐1 human keratinocytes and microvascular endothelial cells, respectively) and were used in testing a chemotherapeutic drug, daunorubicin.

The inert matrices were tolerated well by all studied cell types; however, different changes in morphology and cell‐vitality could be observed. Hydrogels caused the most conspicuous differences in case of A2058 human melanoma cell line. Distinct cell‐assembly and aggregation could be observed in together with the increase in survival rate after Dau treatment. To analyze this phenomenon, an AI‐based application was developed to automatize the microscopic image processing. The application is free to use (https://github.com/enyecz/CancerDetector2).

Data obtained on A2058 cells verified that in optimal hydrogel (15 mol% mPEG), the average area of spheroids are not only maintained, but also even increased after Dau treatment, which would suggest better survival. It was also observed that smaller cellular clusters appeared not long after treatment (3–6 h), but they were later destroyed. Early development of these smaller, short‐lived aggregates/spheroids is probably a response to treatment, but further studies are needed to fully understand the reason behind this phenomenon.

It is also important to highlight the difference between the melanoma cells and the skin‐related normal cells in terms of spheroid formation and resistance against Dau treatment in our PEG‐based hydrogels. The highest survival rate after Dau treatment and in parallel the biggest 3D multicellular aggregates were detected in the case of A2058 melanoma cells. It is possible, that the spheroids, the formation of 3D aggregates by cell–cell interactions, protect against antitumor effects, because the cells in the spheroids are subjected a heterogeneous exposure to antitumor agent [11]. The different spheroid formation ability of our model cells also confirms the previous findings that tumor cells respond to mechanical properties of the microenvironment differently from normal cells [21].

In conclusion, our 3D model and the application may not only be a more reliable model for drug screening assays but may also provide new insights into the mechanisms of tumorigenesis and drug‐resistance.

## Conflict of interest

The authors declare no conflict of interest.

### Peer review

The peer review history for this article is available at https://www.webofscience.com/api/gateway/wos/peer‐review/10.1002/2211‐5463.13719.

## Author contributions

KNE was involved in conceptualization, methodology, investigation, writing—original draft, visualization, and funding acquisition. GE was involved in application development and writing—review & editing. EL was involved in methodology, investigation, writing—original draft, writing—review & editing, and supervision.

## Supporting information


**Appendix S1.** Full description of the synthesis of bis‐triethoxy‐sylilated‐PEG and bis‐dimethyl‐monoethoxy‐sililated PEG monomers.
**Fig. S1.** Cytotoxic effect of daunorubicin (Dau) on A2058 human melanoma cell line.
**Fig. S2.** Cytotoxic effect of daunorubicin (Dau) on HMEC‐1 human dermal microvascular endothelial cell line.
**Fig. S3.** Cytotoxic effect of daunorubicin (Dau) on HaCaT human keratinocyte cell line.
**Fig. S4.** Results of the image analysis of 10% mPEG hydrogels after treatment with daunorubicin (Dau) in comparison with untreated cells.
**Fig. S5.** Results of the image analysis of 15% mPEG hydrogels after treatment with daunorubicin (Dau) in comparison with untreated cells.

**Video S1.** Video showing the effect of daunorubicin in A2058 cell spheroids in 10 mol% mPEG hydrogels.

**Video S2.** Video showing the effect of daunorubicin in A2058 cell spheroids in 15 mol% mPEG hydrogels.

## Data Availability

The data that support the findings of this study are available within the paper and its Supporting Documents. The raw data are available on request from the corresponding author. The final source code off application developed by us and its usage guide can be found at https://github.com/enyecz/CancerDetector2.
