# Supplementary material for: Three‐dimensional, PEG‐based hydrogels induce spheroid formation and enhance viability of A2058 melanoma cells
Source: FEBS Open Bio. 2023 Oct 30;13(12):2356–66. doi: 10.1002/2211-5463.13719 (PMC10699105; doi:10.1002/2211-5463.13719)
Supplement: Supplementary file 1 — Appendix S1. Full description of the synthesis of bis‐triethoxy‐sylilated‐PEG and bis‐dimethyl‐monoethoxy‐sililated PEG monomers. Fig. S1. Cytotoxic effect of daunorubicin (Dau) on A2058 human melanoma cell line. Fig. S2. Cytotoxic effect of daunorubicin (Dau) on HMEC‐1 human dermal microvascular endothelial cell line. Fig. S3. Cytotoxic effect of daunorubicin (Dau) on HaCaT human keratinocyte cell line. Fig. S4. Results of the image analysis of 10% mPEG hydrogels after treatment with daunorubicin (Dau) in comparison with untreated cells. Fig. S5. Results of the image analysis of 15% mPEG hydrogels after treatment with daunorubicin (Dau) in comparison with untreated cells. [file FEB4-13-2356-s002.docx]

**Supplementary Information for**

**Three-dimensional, PEG-based hydrogels induce spheroid formation and enhance viability of A2058 melanoma cells**

Kata Nóra Enyedi^1,2^, Gábor Enyedi^3^, Eszter Lajkó^4*^

^1^ Eötvös Loránd University, Faculty of Science, Institute of Chemistry, Budapest, Hungary

^2^ ELKH-ELTE Research Group of the Peptide Chemistry Institute, Department of Organic Chemistry, Eötvös Loránd University, Budapest, Hungary

^3^ En-Co Software Zrt., Department of Research and Development, Budapest, Hungary

^4^ Department of Genetics, Cell and Immunobiology, Semmelweis University, Budapest, Hungary

*Corresponding Author: Eszter Lajkó

e-mail: lajko.eszter@med.semmelweis-univ.hu

Postal address: H-1089 Hungary, Department of Genetics, Cell and Immunobiology, Semmelweis University, Nagyvárad tér 4.

PEG 2000 was purchased from Sigma-Aldrich. Alkoxy-silanes were from Fluorochem Limited. NaF and anhydrous methyl-tert-butyl-ether (MTBE) and n-hexane were purchased from VWR international. All reagents and solvents were used without further purification. When reactions required anhydrous conditions, dry solvents were used.

NMR spectra was recorded on a Bruker Ascend 400 spectrometer. Chemical shifts (δ) are reported in parts per million (ppm), Signals are indicated as s (singlet), d (doublet), t (triplet), q (quartet), dt (double triplet), m(multiplet).

*Synthesis of bis-tosylated PEG*

The synthesis was performed according to the method described previously [[1](#_ENREF_1)].

PEG 2000 (1 eq, 10 mmol, 20 g), *p*-toluenesulfonyl chloride (9 eq, 90 mmol, 17.1 g) was dissolved in CH_2_Cl_2_ (100 mL). Triethylamine (9 eq, 90 mmol, 12.5 mL) and DMAP (5 mol %, 0.5 mmol, 61 mg) were added. After stirring for 48 h at R.T., the solution was filtered, and the reaction mixture was then washed with sat. sat. NH_4_Cl three times. The organic layer was separated, dried over Na_2_SO_4_ and concentrated *in vacuo*. The resulting brown oil purified on a silica pad, using n-hexane:EtOAc (1:1) to remove impurities, then washed with CHCl_3_:MeOH (4:1) and concentrated *in vacuo.* The resulting brownish oil was cooled in a freezer to help solidify. Rubbing the crude product with dry MTBE bis-tosylated PEG as yellowish-white powder (7.8 mmol, 18.1 g, 78%).

**^1^H-NMR (400 MHz, CDCl_3_):** *δ* 7.79 (d, 4H, J = 6.6 Hz), 7.34 (d, 4H, J = 6.6 Hz), 4.13 (m, 4H), 3.62 (board, 174H, C*H*_2_-O), 2.46 (s, 6H).

**^13^C-NMR (400 MHz, CDCl_3_):** *δ* 144.72, 133.11, 129.79, 127.95, 70.56 (broad), 69.20, 68.67, 21.60.

*Synthesis of bis-triethoxy-silylated PEG monomer (tPEG)*

The Bis-silylated PEG was synthesised according to the protocol previously described by Hu et al. [[2](#_ENREF_2)].

Bis-tosylated-PEG was (2.3 g, 1.1 mmol, 1 eq) was dissolved in anhydrous MeTHF (24 mL) under argon. 3-aminoropyltriethoxysilane (1.55 µL, 6.6 mmol, 6 eq) were added. The mixture was kept at reflux for 48 h. Then the solvents were removed under vacuum and the reaction mixture was precipitated in hexane. The resulting white solid was washed with anhydrous MTBE 3 times, followed with hexane 3 times, and then vacuum dried. The bis-silylated PEG was then stored in a desiccator, over P2O5, under vacuum, at room temperature.

**^1^H-NMR (400 MHz, CDCl_3_):** δ 3.8 (q, 12H, J = 8.0 Hz), 3.64 (board, 174H), 2.80 (t, 4H, J=8.0 ), 2.64 (t, 4H, J=8.0) (t, 4H, J=8,0 ) 1.60 (q, 4H, J= 8.0),1.21 (t, 18H, J = 8.0 Hz), 0.62 (m, 4H)

**^13^C-NMR (400 MHz, CDCl_3_):** *δ* 70.39(broad), 69.70, 57.90, 48.83, 43.04, 22.90, 18.12, 7.73.

*Synthesis of bis-dimethyl-monoethoxy-silylated PEG monomer (mPEG)*

The Bis-silylated PEG was synthesised according to the protocol previously described by Hu et al. [[2](#_ENREF_2)].

Bis-tosylated-PEG was (1.14 g, 0.54 mmol) was dissolved in anhydrous MeTHF (24 mL) under argon. 3-aminoropyldimethyl-ethoxysilane (610 µL, 3.24 mmol, 6 eq) were added. The mixture was kept at reflux for 48 h. Then the solvents were removed under vacuum and the reaction mixture was precipitated in hexane. The resulting white solid was washed with anhydrous MTBE 3 times, followed with hexane 3 times, and then vacuum dried. The bis-silylated PEG was then stored in a desiccator, over P2O5, under vacuum, at room temperature.

**^1^H-NMR (400 MHz, CDCl_3_):** δ 3.8 (q, 4H, J = 8.0 Hz), 3.64 (board, 174H), 2.80 (t, 4H, J=8.0 ), 2.64 (t, 4H, J=8.0) (t, 4H, J=8,0 ) 1.60 (q, 4H, J= 8.0), 1.22 (t, 6H, J = 8.0 Hz), 0.64 (m, 4H), -0.01 (m, 12H)

**13C NMR (400 MHz, CDCl3):** *δ* 70.56 (broad), 70.02, 57.95, 48.96, 43.30, 25.75, 18.12, 13.7, -2.46

*Cytotoxicity of daunorubicin (Dau) in 2D cells*


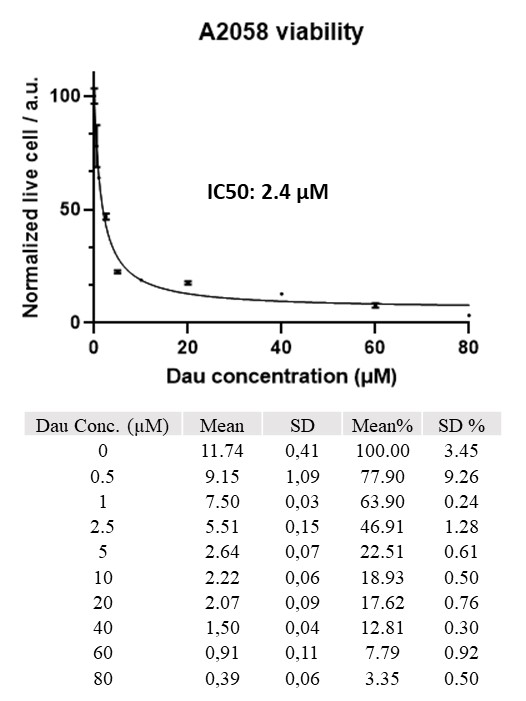


Supplementary Fig S1. Cytotoxic effect of daunorubicin (Dau) on A2058 human melanoma cell line

The data are normalized to the control wells. The IC50 value of Dau was determined by fitting a sigmoidal dose-response curve to the data, using OriginPro 2020. Data are given as mean values ± standard deviation (SD), (n=3).


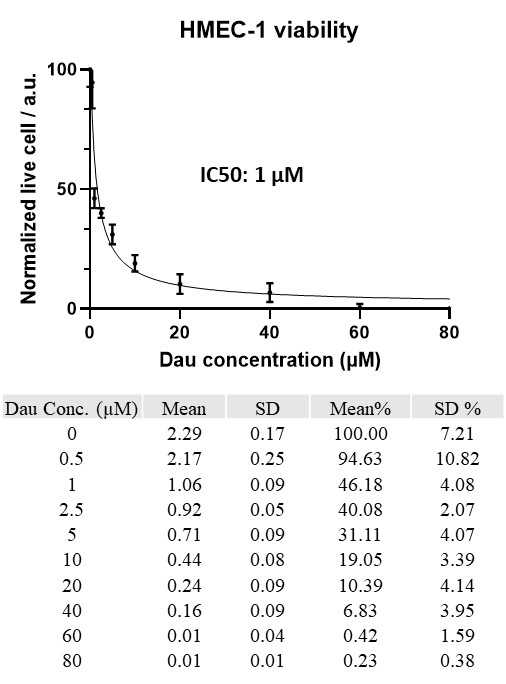


Supplementary Fig S2. Cytotoxic effect of daunorubicin (Dau) on HMEC-1 human dermal microvascular endothelial cell line

The data are normalized to the control wells. The IC50 value of Dau was determined by fitting a sigmoidal dose-response curve to the data, using OriginPro 2020. Data are given as mean values ± standard deviation (SD), (n=3).


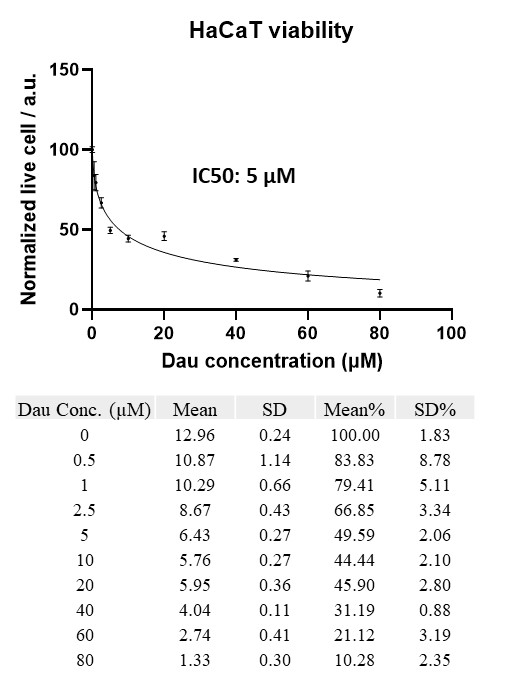


Supplementary Fig S3. Cytotoxic effect of daunorubicin (Dau) on HaCaT human keratinocyte cell line

The data are normalized to the control wells. The IC50 value of Dau was determined by fitting a sigmoidal dose-response curve to the data, using OriginPro 2020. Data are given as mean values ± standard deviation (SD), (n=3).





*Supplementary Fig S4. Results of the image analysis of 10 mol% mPEG hydrogels after treatment with daunorubicin (Dau) in comparison with untreated cells. Images were taken each hour and terminated when no significant changes can be registered further (22h). A) Number of spheroids and B) average area of spheroids in 10 mol% mPEG hydrogels after Dau treatment. Data are presented as mean values ± standard deviation (SD) from three independent experiments. The levels of significance were determined by paired, two-tailed T-test if the values of a given data series were compared to the 0h time point. Unpaired two-tailed T-test was used to determine significance if at given time points the values of the two hydrogels were compared. Values of p < 0.05 were considered significant compared to 1h indicated by * asterisks and between hydrogels indicated by #.*

*

*

*Supplementary Fig S*5. *Results of the image analysis of 15 mol% mPEG hydrogels after treatment with daunorubicin (Dau) in comparison with untreated cells. Images were taken each hour and terminated when no significant changes can be registered further (22h). A) Number of spheroids and B) average area of spheroids in 15 mol% mPEG hydrogels after Dau treatment. Data are presented as mean values ± standard deviation (SD) from three independent experiments. The levels of significance were determined by paired, two-tailed T-test if the values of a given data series were compared to the 0h time point. Unpaired two-tailed T-test was used to determine significance if at given time points the values of the two hydrogels were compared. Values of p < 0.05 were considered significant compared to 1h indicated by * asterisks and between hydrogels indicated by #.*

**References**

1. Pierre-Edouard, D, Damien, W, Francine, C-D and François, D (2012) Ultrasound-promoted tosylation of oligo(ethylene glycols). *Ultrason Sonochem*, **19**: 1201-1204.

2. Hu, S, Sun, L, Liu, M, Hongda, Z, Guo, H, Sun, H and Sun, H (2015) A highly dispersible silica pH nanosensor with expanded measurement ranges. *New J Chem*, **39**.
